# Connexin-43 reduction prevents muscle defects in a mouse model of manifesting Duchenne muscular dystrophy female carriers

**DOI:** 10.1038/s41598-020-62844-9

**Published:** 2020-03-30

**Authors:** Julie Nouet, Eric Himelman, Kevin C. Lahey, Qingshi Zhao, Diego Fraidenraich

**Affiliations:** Department of Cell Biology and Molecular Medicine, Rutgers Biomedical and Health Sciences, New Jersey Medical School, Newark, NJ USA

**Keywords:** Cardiomyopathies, Molecular medicine

## Abstract

Duchenne muscular dystrophy (DMD) is a severe X-linked neuromuscular disorder that affects males. However, 8% of female carriers are symptomatic and underrepresented in research due to the lack of animal models. We generated a symptomatic mouse model of DMD carriers via injection of *mdx* (murine DMD) embryonic stem cells (ESCs) into wild-type (WT) blastocysts (*mdx*/WT chimera). *mdx*/WT chimeras developed cardiomyopathic features and dystrophic skeletal muscle phenotypes including elevated mononuclear invasion, central nucleation, fibrosis and declined forelimb grip strength. The disease was accompanied by connexin-43 (Cx43) aberrantly enhanced in both cardiac and skeletal muscles and remodeled in the heart. Genetic reduction of Cx43-copy number in *mdx*/WT-Cx43(+/−) chimeras protected them from both cardiac and skeletal muscle fiber damage. In dystrophic skeletal muscle, Cx43 expression was not seen in the fibers but in adjacent F4/80+ mononuclear cells. Ethidium Bromide uptake in purified F4/80+/CD11b+ *mdx* macrophages revealed functional activity of Cx43, which was inhibited by administration of Gap19 peptide mimetic, a Cx43 hemichannel-specific inhibitor. Thus, we suggest that Cx43 reduction in symptomatic DMD carrier mice leads to prevention of Cx43 remodeling in the heart and prevention of aberrant Cx43 hemichannel activity in the skeletal muscle macrophages neighboring Cx43 non-expressing fibers.

## Introduction

Duchenne muscular dystrophy (DMD) is an incurable X-linked recessive disorder characterized by mutations in the dystrophin gene. The dystrophin protein is an essential part of the dystrophin-glycoprotein complex (DGC), which provides structure and mechanically-induced signal propagation between intracellular and extracellular environments^1^. X-linked disorders only affect male patients; however, a percentage of female DMD heterozygotes are symptomatic. Symptomatic female carriers consist of about 8–40% of definite DMD carriers and begin to present symptoms between the ages of 2–47 years old^2,3^. Patients display a range of severe to less severe phenotypes. It is generally accepted to affect 8% of female carriers, yet this statistic aligns with those affected by dilated cardiomyopathy. A higher percentage (17%) suffer from muscle weakness^3–10^. Heart complications can be further expanded to include left-ventricle dilation (40%)^10^.

Research on symptomatic carriers has been confined to clinical observations largely due to the absence of a representative animal model^2,3,9,11,12^. These epidemiological studies are subject to a larger margin of error from confounding variables, necessitating a DMD animal model that successfully recapitulates the symptomatic carrier phenotypes and mosaicism. X-linked muscular dystrophy mice (*mdx*)^13^ heterozygotes, *mdx*(+/−), show normal muscle histology and do not develop cardiomyopathy^11,12^. Recently, we described the generation of a model to study symptomatic DMD carriers that faithfully recapitulates the pathology and symptoms (*mdx*/WT mouse chimeras)^14^. Symptomatic *mdx* mouse chimeras were generated via injection of *mdx* embryonic stem cells (ESCs) into wild-type (WT) blastocysts. *Mdx*/WT chimeras feature patches of dystrophin negative(−) and dystrophin positive(+) cells^14^. This mosaic model resembles well-documented dystrophin mosaicism in human female DMD carriers due to randomized X-inactivation^14,15^.

Cardiomyopathy and arrhythmias are tightly correlated with dysregulation of gap junction (GJ) transmembrane protein connexin-43 (Cx43) based on its critical role in synchronous electrical impulse propagation between ventricular contractile cardiomyocytes^16–19^. Cx43 is increasingly recognized as being pathologically disrupted in ischemia leading to cell death and tissue damage, and in Duchenne muscular dystrophy associated cardiomyopathy^20–23^. *Mdx* hearts display increased protein levels and pathological remodeling of Cx43 away from the cell-to-cell junction intercalated disc (ID) compared to wild-type (WT) mice^16,24,25^. Cx43 hemichannel remodeling to the lateral side of the cardiomyocyte and internalization leads to electrical uncoupling and arrhythmogenesis^26^. Normalization of Cx43 levels through genetic reduction in *mdx*:Cx43(+/−) mice prevented Cx43 mislocalization to the lateral borders as well as cardiac dysfunction in aged *mdx* mice^16,24^. Reduction of Cx43 hemichannel activity through treatment with Gap19 selective peptide mimetic inhibitor, improved myocardial ischemia, reperfusion, and cardiac damage by lowering the occurrence of arrhythmias, metabolic injury, and mortality^16,23,27^. Pathology of *mdx* cardiomyopathy can be attenuated by preventing lateralized Cx43 hemichannels or blocking hemichannel activity^28,29^.

The role of Cx43 in skeletal muscle pathology is less known. In contrast to cardiac muscle that requires constant GJ intracellular communication between cardiomyocytes to synchronize heart contraction, mature skeletal muscle cells fuse to form a syncytium, therefore the formation of GJs is unnecessary in mature muscle fibers. Expression of Cx43 is absent in control, uninjured tissues, and increases after denervation or injury and can be seen as punctate staining; however, it does not overlap with muscle stem cell marker (Pax7+)^30,31^. Cx43 becomes dysregulated in many injured or infectious environments including ischemia/reperfusion injury, denervation, and infection/sepsis^31–33^. In *mdx* mice, Cx43 has been associated with apoptosis and necrosis in myofibers^34^. As the role of Cx43 in cardiac myocytes became clear in DMD patients, we sought to investigate the understudied pathological role of Cx43 in the skeletal muscle. In the present study, we explore a rescue role of Cx43 reduction in both the cardiac and skeletal muscle of *mdx*/WT chimeras.

## Results

### Genetic reduction of Cx43 rescues the *mdx*/WT-Cx43(+/−) chimeric heart

**Figure 1:**
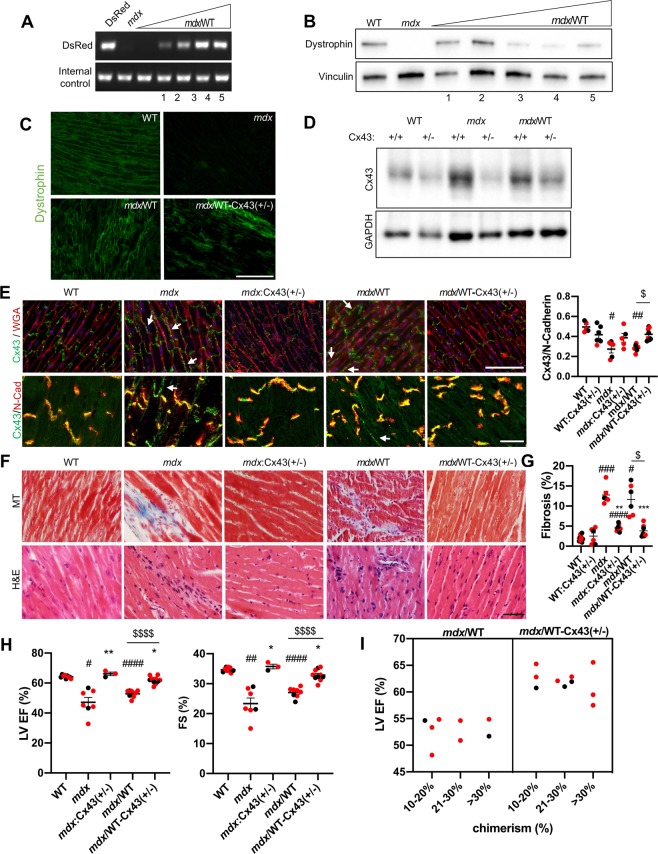
Cx43 copy number reduction rescues *mdx* symptomatic carrier mouse hearts. (**A**) A representative agarose gel shows PCR products of the DsRed transgene (top band) from cardiac genomic DNA of DsRed (100% control), *mdx* (0% control) and *mdx*/WT mice with increasing degrees of chimerism (left to right). Internal control (bottom band) serves as a normalizer. **(B)** A representative western blot stained for dystrophin (top band), using cardiac protein extracts of WT (100% control), *mdx* (0% control) and *mdx*/WT mice with decreasing levels of dystrophin (left to right). Vinculin (bottom band): internal control. Samples presented in A (1–5) and B (1–5) are paired. **(C)** Representative immunohistochemistry of ventricular cryosections stained for dystrophin (green). WT control is dystrophin+ and mdx control is dystrophin**–**. Magnification: 200X. Scale bar: 150 μm. **(D)** Representative western blot stained for Cx43 (top band) using cardiac protein extracts. GAPDH (bottom band): internal control. **(E)** Top row: representative immunofluorescence images of ventricular cryosections stained for Cx43 (green), along with wheat germ agglutinin (WGA) (red) to demarcate cell borders. White arrows point to Cx43 lateralization (remodeling) in mdx mice and *mdx*/WT chimeras. Magnification: 200x. Scale bar: 150 µm. Bottom row: representative images for Cx43 and intercalated disc marker N-Cadherin (N-Cad). Magnification: 600x. Scale bar: 25 µm. Cx43 retention to the N-Cad is quantified in graph right. Sample size: respectively left to right N(5,6,5,5,7,8). **(F)** Representative Masson trichrome (MT) (top row) and H&E (bottom row) images show fibrosis (MT) and mononuclear invasion (H&E) in *mdx* and *mdx*/WT but not in WT, WT:Cx43(+/−), *mdx*:Cx43(+/−) mice and *mdx*/WT-Cx43(+/−) chimeras. Magnification: 400x. Scale bar: 100 µm. **(G)** Quantification of fibrosis from (**F)** (MT). N(10,6,6,9,7,8). **(H)** Left ventricle (LV) ejection fraction (%EF) and fractional shortening (%FS) show decreased function in *mdx* mice and *mdx*/WT chimeras relative to WT, *mdx*:Cx43(+/−) mice and *mdx*/WT-Cx43(+/−) chimeras. Sample size: N(10,6,6,9,8,9);. **(I)** LV EF(%) in *mdx*/WT and *mdx*/WT-Cx43(+/−) chimeras with range of chimerism: 10–20%, 21–30%, >31%. N(8,9). See *Statistics*: ^#^P < 0.05 versus WT, *P < 0.05 versus *mdx*, ^$^P < 0.05 *mdx*/WT versus *mdx*/WT-Cx43(+/−) chimeras. Red dots represent female mice. Mouse aged 10–14 months. Uncropped gel and blots are displayed in Supplementary Fig. S6.

**Figure 2:**
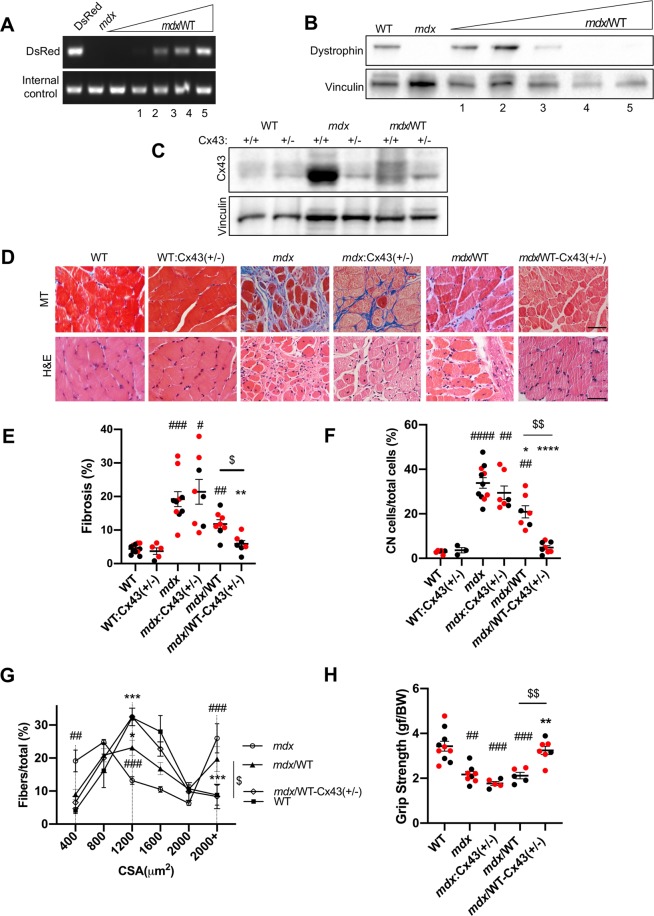
Cx43 copy number reduction rescues *mdx*/WT diaphragm muscle. (**A**) A representative agarose gel shows PCR products of the DsRed transgene (top band) from diaphragm genomic DNA of DsRed (100% control), *mdx* (0% control) and *mdx*/WT chimeras with increasing degrees of chimerism (left to right). Internal control (bottom band) serves as a normalizer. **(B)** Representative western blot stained for dystrophin (top band), using diaphragm protein extracts of WT (100% control), *mdx* (0% control) and *mdx*/WT chimeras with decreasing levels of dystrophin (left to right). Vinculin (bottom band): internal control. Samples presented in A (1–5) and B (1–5) are paired. **(C)** Representative western blot stained for Cx43 (top band) using diaphragm protein extracts of WT, WT:Cx43(+/−), *mdx*, *mdx*:Cx43(+/−) mice, *mdx*/WT and *mdx*/WT-Cx43(+/−) chimeras. Vinculin (bottom band): internal control. Sample size: WT, WT:Cx43(+/−), *mdx*, *mdx*:Cx43(+/−), *mdx*/WT, *mdx*/WT-Cx43(+/−) respectively left to right N(7,7,5,8,9). **(D)** Representative Masson trichrome (MT) (top row) and H&E (bottom row) images show fibrosis (MT), mononuclear invasion (H&E) and central nucleation (H&E) in *mdx*, *mdx*:Cx43(+/−) mice and *mdx*/WT chimeras but not in WT, WT:Cx43(+/−) mice and *mdx*/WT-Cx43(+/−) chimeras. Magnification: 400×. Scale bar: 100 μm. **(E)** Quantification of fibrosis (%). Sample size: respectively left to right N(11,5,10,8,8,7). **(F)** Quantification of central nucleation (D, bottom). N(6,3,11,7,7,8). **(G)** Distribution profile of cross-sectional fiber area (CSA) of WT, *mdx*, *mdx*/WT and *mdx*/WT-Cx43(+/−) chimeric diaphragm muscle throughout the 400–2000+ µm^2^ range. Samples: WT, *mdx*, *mdx*/WT, and *mdx*/WT-Cx43(+/−) N(3,5,5,5) respectively. Statistics (Two-way ANOVA and Tukey’s multiple comparisons test) were run for fibers of low caliber 400 µm^2^, middle 1200 µm^2^, and high 2000+ µm^2^. **(H)** Grip strength was determined in WT, *mdx*, *mdx*:Cx43(+/−) mice, *mdx*/WT and *mdx*/WT-Cx43(+/−) chimeras. N(10,8,5,5,7); experiments were performed in triplicate. See *Statistics*: ^#^p < 0.05 versus WT, ^*^P < 0.05 versus *mdx*, ^$^P < 0.05 *mdx*/WT versus *mdx*/WT-Cx43(+/−) chimeras. Red dots represent female mice. Mouse aged: 10–14 months. Uncropped gel and blots are displayed in Supplementary Fig. S6.

We generated *mdx*/WT and *mdx*/WT-Cx43(+/−) chimeras by injecting *mdx* (dystrophin−) murine embryonic stem cells (ESCs) into WT:Cx43(+/+) and WT:Cx43(+/−) (dystrophin+) blastocysts^14^. Seven day-old pups were genotyped for the presence of a heterozygous mutation in the Cx43 gene using DNA from tail tips. Half of the littermates contained the mutation and half did not (data not shown). *Mdx* ESCs contain a reporter DsRed transgene inserted into their genome whose ratio of band intensities compared to internal control, supplied by Jax labs, was used to determine the degree of ESC-derived cells (degree of chimerism)^14^. The presence of the DsRed transgene was assessed in pups tail tips (data not shown)^14^ and then confirmed at time of sacrifice using heart (Fig. 1A), diaphragm (Fig. 2A), and pectoralis (Supplementary Fig. S3). Tissue from mice transgenic for DsRed and *mdx* mice were used as a positive (100%) control and a negative (0%) control, respectively. Mouse chimeras in the range of 10–30% (DsRed) contained 10–30% of ESC-derived cells and 90–70% of blastocyst-derived cells respectively. Results obtained from DsRed inversely correlated with those obtained by western blot analysis of the dystrophin protein in heart, diaphragm, and pectoralis extracts (Figs. 1B and 2B, Supplementary Fig. S3). Quantification of dystrophin in *mdx*/WT and *mdx*/WT-Cx43(+/−) chimeras compared to WT (positive control) and *mdx* (negative control) showed a range of dystrophin expression (Supplementary Fig. S1). The presence of dystrophin+ and dystrophin− patches in the heart were observed in both *mdx*/WT and *mdx*/WT-Cx43(+/−) chimeras by immunofluorescence detection in cardiac cryosections (Fig. 1C). Variations in dystrophin levels became evident when comparative analysis was conducted across individual chimeras (Supplementary Fig. S1). For example, chimera 1 showed similar levels of dystrophin expression in heart, diaphragm, and pectoralis, but chimera 4 showed higher levels of dystrophin expression in heart than those in diaphragm or pectoralis.

Cx43 protein reduction in the heart and skeletal muscle of *mdx*:Cx43(+/−) mice and *mdx*/WT-Cx43(+/−) chimeras was confirmed through Cx43 immunoblotting (Figs. 1D and 2C). Quantification of Cx43 showed significantly higher Cx43 levels in *mdx* and *mdx*/WT with respect to WT, *mdx*:Cx43(+/−), *mdx*/WT-Cx43(+/−) despite variations in the degree of chimerism (Supplementary Fig. S1).

To study the pattern of Cx43 expression we performed immunofluorescence analysis. Cx43 was confined to the intercalated disc (ID) of the cardiomyocytes in WT, *mdx*:Cx43(+/−) mice and *mdx*/WT-Cx43(+/−) chimeric heart sections (Fig. 1E). Cx43 was additionally detected on the lateral cell borders (see white arrows) of cardiomyocytes in *mdx* hearts and *mdx*/WT chimera hearts (Fig. 1E). Cx43 retention to the ID, marked by N-Cadherin (N-Cad), was quantified (Fig. 1E-right). This suggests that Cx43 remodeling (lateral distribution) is prevented in the heart of both *mdx*:Cx43(+/−) mice and *mdx*/WT-Cx43(+/−) chimeras. Furthermore, evidence of fibrosis (Masson trichrome, MT) and mononuclear invasion (H&E) was observed in aged (10–14 months) *mdx* mice and *mdx*/WT chimeric hearts but not in WT, WT:Cx43(+/−), *mdx*:Cx43(+/−) mice and *mdx*/WT-Cx43(+/−) chimeric hearts (Fig. 1F,G). Lower magnifcation photos have been provided in Supplementary Fig. S2. This suggests that lack of Cx43 remodeling as the result of Cx43 reduction in *mdx* and chimeric mice may prevent cardiac histopathology. In line with this, restored cardiac function left ventricle (LV) ejection fraction (%EF) and fractional shortening (%FS) was observed in *mdx*:Cx43(+/−)^24^ mice and *mdx*/WT-Cx43(+/−) chimeras (Fig. 1H,I). Altogether, the results suggest that genetic reduction of Cx43, Cx43(+/−), prevents muscular dystrophy associated cardiomyopathy in the heart of manifesting DMD carrier mice.

### Genetic reduction of Cx43 rescues the *mdx*/WT-Cx43(+/−) chimeric skeletal muscle

We also studied the dystrophic skeletal muscle in chimeric mice to test a potential rescue role of genetic Cx43 reduction. We focused primarily on the diaphragm as it is the most affected skeletal muscle tissue in DMD patients and *mdx* mice^35,36^ and confirmed our observations, with similar results, in the pectoralis muscle (Supplementary Figs. S2 and S3). Histopathological analysis revealed augmented fibrosis (MT), central nucleation and mononuclear invasion (H&E) in the diaphragm muscle of *mdx* and *mdx*:Cx43(+/−) mice, relative to that of WT mice (Fig. 2D–F). This suggests that genetic reduction of Cx43 does not benefit the *mdx* skeletal muscle. However, fibrosis, mononuclear infiltration, and central nucleation were reduced in the diaphragm muscle of *mdx*/WT-Cx43(+/−) chimeras relative to that of *mdx*/WT chimeras (Fig. 2D–F). This suggests that genetic reduction of Cx43 prevents pathology in the skeletal muscle of the *mdx*/WT-Cx43(+/−) chimeras. Accordingly, the skeletal muscle fiber cross-sectional area (CSA) distribution profile was assessed. While most of the fibers in WT diaphragm were in the range of 1,200–1,600 µm^2^, fibers in mdx diaphragm were in a broader range, spanning from 400 µm^2^ to 2,000 µm^2^ at the expense of a reduced number of fibers in the 1,200–1,600 µm^2^ range*. mdx*/WT-Cx43(+/−) chimeric diaphragm muscle was alike to that of WT, in which most fibers were in the range of 1200–1600 µm^2^ (Fig. 2G). *mdx/*WT had less medium caliber (1,200–1,600 µm^2^), and more high caliber (2000+ µm^2^) fibers, shifting towards the *mdx* phenotype (Fig. 2G). Lastly, grip strength was improved in *mdx*/WT-Cx43(+/−) chimeras but not in *mdx*/WT chimeras or *mdx*:Cx43(+/−) mice relative to WT mice (Fig. 2H). Taken all together, the results suggest that genetic reduction of Cx43 corrects muscular dystrophy in DMD carrier mice, but not in DMD mice.

### Cx43 is enhanced and functional in *mdx* but not in WT skeletal muscle macrophages

**Figure 3:**
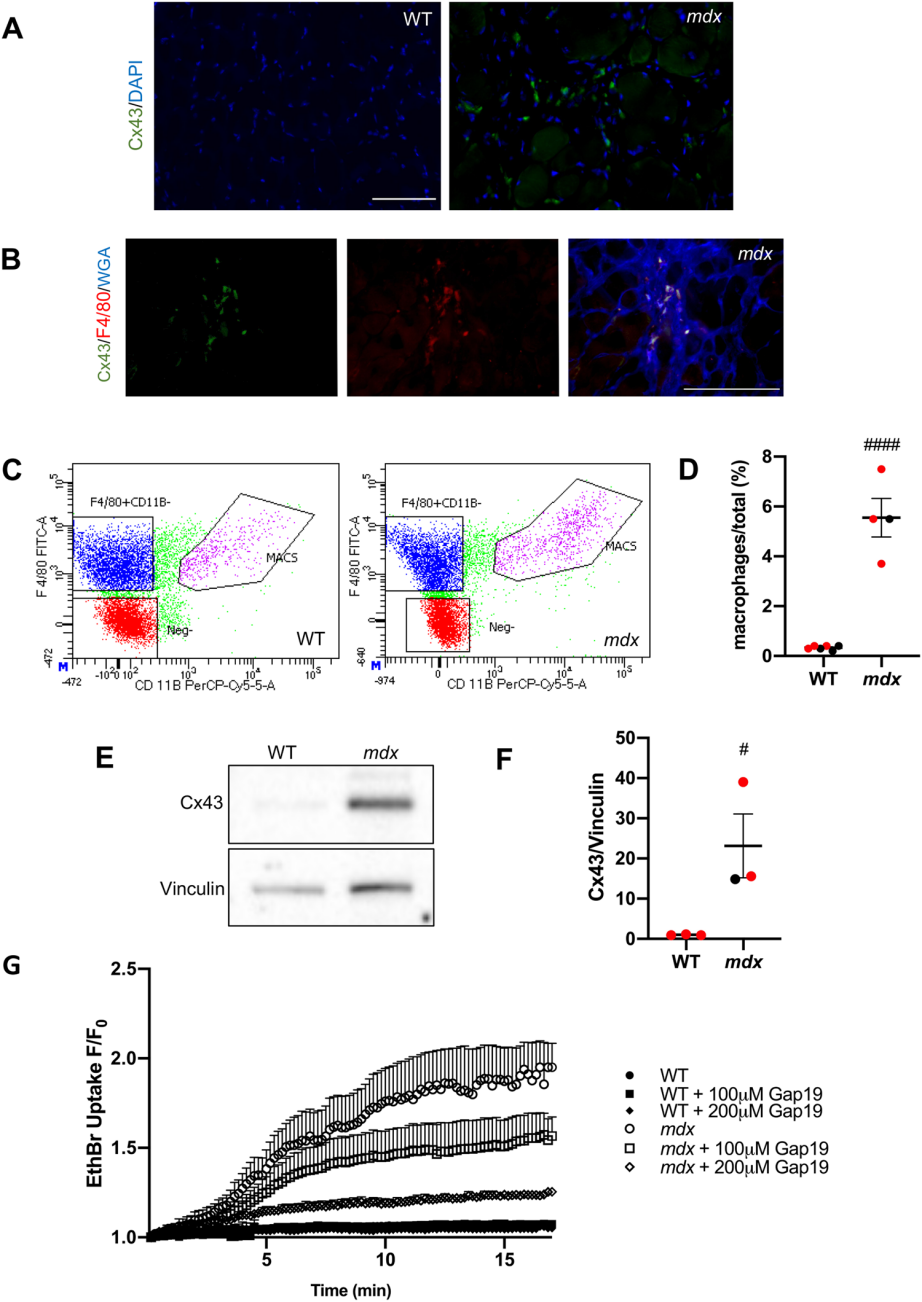
Cx43 is enhanced and functional in *mdx* but not in WT skeletal muscle macrophages. **(A)** Representative immunofluorescence images of Cx43 (green) and DAPI (blue) show the presence of Cx43 in cryosections of *mdx* but not of WT diaphragm muscle. Magnification: 200x. Scale Bar: 150 μm. **(B)** Representative immunofluorescent images show overlap of Cx43 (green), F4/80 (red), and WGA (blue) in cryosections of *mdx* diaphragm muscle. Magnification: 200x. Scale Bar: 150 μm. **(C)** Representative output of FACS-sorted macrophages (MACS) from WT (left) and *mdx* (right) diaphragm muscle. **(D)** Quantification of percent F4/80+/Cd11b+ cells versus total shows significantly more macrophages in *mdx* relative to WT diaphragm. F4/80+/Cd11b+ MACS were used for subsequent experiments indicated in (**E**,**G**). Number of mice: N(6, 4). **(E)** Representative western blot analysis and **(F)** its quantification (N = 3) using an antibody reactive with Cx43 (top). Vinculin (bottom): internal control. **(G)** Ethidium Bromide (EthBr) uptake ± Gap19 peptide mimetic (0 µM, 100 µM or 200 µM) Cx43-selective hemichannel inhibitor in isolated F4/80+/Cd11b+ macrophages from WT or *mdx* diaphragms. Values were collected 30 s after EthBr addition and reported as F/F_0_. See *Statistics*: ^#^P < 0.05 versus WT. Red dots represent female mice. Uncropped blots are displayed in Supplementary Fig. S6.

Our results suggest that Cx43 is enhanced in the skeletal muscle of *mdx* mice and *mdx*/WT chimeras relative to WT mice (Fig. 2C and Supplementary Fig. S1). Our results also suggest that Cx43 reduction prevents the development of skeletal muscle dystrophy in *mdx*/WT-Cx43(+/−) chimeras, but not in *mdx*:Cx43(+/−) mice (Fig. 2D–H). This finding in the skeletal muscle contrasts with that in the heart, where Cx43 reduction leads to corrections in both *mdx*/WT-Cx43(+/−) chimeras and *mdx*:Cx43(+/−) mice hearts (Fig. 1F–I). To begin to reconcile this dissimilar role of Cx43, we examined the Cx43 pattern of expression in skeletal muscle by immunofluorescence. We did not detect Cx43 on any part of the *mdx* or WT mature skeletal muscle fibers (Fig. 3A,B). This suggests that there may be other cell types distinct from the fibers that express Cx43 in the skeletal muscle, particularly in dystrophic mice, because Cx43 was seemingly in areas occupied by mononuclear cells (Fig. 3A,B). Due to previous reports of Cx43 in macrophages^37,38^ we performed immunofluorescent analysis for Cx43 and F4/80 macrophage marker. This revealed that Cx43 was detected only in *mdx* F4/80+ mononuclear cells in the interstitial space between skeletal muscle fibers, areas where mononuclear invasion occurs (Fig. 3B).

To further investigate the role of Cx43 in DMD skeletal muscle macrophages, macrophage-enriched FACS-sorted F4/80+/Cd11b+ cells were purified from *mdx* and WT diaphragms (Fig. 3C,D). We obtained a higher percentage of the macrophage-enriched F4/80+/Cd11b+ population in *mdx* diaphragms relative to WT diaphragms (Fig. 3C,D). Furthermore, Cx43 protein expression in the F4/80+/Cd11b+ macrophages was enhanced in *mdx* versus WT (Fig. 3E,F).

To test the activity of Cx43 hemichannels in diaphragm F4/80+/Cd11b+ cells, we performed Ethidium Bromide (EthBr) uptake assays (Fig. 3G). Gap19 was used as a Cx43 hemichannel-specific peptide mimetic inhibitor. *Mdx* F4/80+/Cd11b+ cells exhibited a faster rate of EthBr uptake relative to WT F4/80+/Cd11b+ cells (Fig. 3G). However, Gap19 administration resulted in a dose-dependent slowing of EthBr uptake activity in *mdx* F4/80+/Cd11b+ cells, with negligible impact on WT F4/80+/Cd11b+ cells (Fig. 3G).

Because cardiac macrophages were also observed in the dystrophic heart, we performed F4/80 and Cx43 immunofluorescence in WT and *mdx* hearts to study a potential overlap. However, unlike the presence of overlap in the dystrophic diaphragm (Fig. 3B), there was no co-staining in the heart (Supplementary Fig. S4). Furthermore, macrophage-enriched FACS-sorted F4/80+/Cd11b+ cells purified from *mdx* hearts revealed negligible detection of Cx43 protein expression. Gravity-settled *mdx* heart myocytes were used as a Cx43 positive control (Supplementary Fig. S4). Altogether, the results open the possibility that detrimental activity of enhanced Cx43 hemichannels from resident skeletal muscle macrophages may be mitigated by Cx43 reduction in a dystrophic, chimeric environment.

## Discussion

In this study, we show that Cx43-copy number reduction in *mdx*/WT-Cx43(+/−) chimeras result in a rescue of DMD pathology in both the heart and the skeletal muscle. These results are aligned with previous studies in the heart, in which normalized Cx43 levels benefit *mdx* cardiac pathology^16,24^; but are unlike the observations in *mdx* skeletal muscle, in which normalized Cx43 levels do not benefit skeletal muscle pathology. We suggest aberrant Cx43 hemichannel activity in *mdx* macrophages promotes the dystrophic phenotype in the skeletal muscle. We also suggest the lack of rescue in *mdx*:Cx43(+/−) skeletal muscle compared to *mdx*/WT-Cx43(+/−) chimeras can be explained by the presence of some dystrophin in the symptomatic DMD carrier and the lack of dystrophin in mdx:Cx43(+/−) mice. Complete absence of dystrophin in mdx:Cx43(+/−) skeletal muscle may lead to exacerbation of a more oxidative environment that may ultimately impinge on the state of the macrophages. Overall, we propose symptomatic *mdx* carrier skeletal muscle, in addition to the heart, benefit from Cx43 normalization in the presence of some dystrophin+ fibers.

DMD patients exhibit chronic inflammation of the heart and skeletal muscle in the form of fibrosis, necrosis, and functional deficiencies. In *mdx* mice there is an upregulation of Cx43 in the affected muscles, possibly to compensate for increased injury, satellite cell exhaustion and increased myotube regeneration of the muscle^39–43^. Similar to the heart, Cx43 is required for myoblast differentiation *in vitro*^44–46^. In contrast with the heart where gap junction intracellular communication (GJIC) is essential, mature skeletal muscle cells fuse to form a syncytium, therefore they do not rely on GJ formation^44,45^. Although absent from control, basal tissues Cx43 is expressed post-muscle injury and can be seen as punctate staining^30,31^. Under similar circumstances including ischemia/reperfusion injury, denervation, and infection/sepsis, this study supports that macrophages upregulate Cx43^31–33^. Despite the different roles of Cx43 in response to cardiac and skeletal muscle damage, higher expression is correlated to enhanced cell death^34,47,48^.

Current treatments only provide short-term relief for DMD patients and carriers, although promising findings have been shown to attenuate the inflammatory phenotype. One of the most commonly prescribed treatments, Dexamethasone (DEX), is a glucocorticoid used to build strength and muscle. However, DEX has led to an increase in Cx43 hemichannels that can exacerbate skeletal muscle atrophy^49^. In sepsis, Cx43 normalization via deletion or pharmacological inhibition is associated with a reduction of secreted necrotic factors, including cytokines and ATP, thereby increasing survival^38^. Peptide mimetics, like Gap19, have been successful because they are capable of inhibiting Cx43 hemichannel dysregulation, but maintain gap junction function in other cell types^37^.

In *mdx* mice, Cx43 is expressed in between mature skeletal muscle fibers, where lymphocytes, neutrophils, and macrophages reside. However, lymphocytes and neutrophils do not express Cx43^38^. Macrophages also have the capability to fuse with adjacent fibers, which may contribute to DMD pathology^37,46,50,51^. Aberrant hemichannel activity has also been observed in *mdx* mouse myofibers to cause apoptosis^22,33,48,52^. Based on localization of Cx43 to the mononuclear cells, we postulate that reduction of hemichannel activity in skeletal muscle macrophages is linked to decreased cell death, suppressed activation of pro-inflammatory factors, and therefore, slowed muscle deterioration^17,34,53^.

In our mouse model of symptomatic DMD carriers, we have shown that Cx43 has divergent roles in skeletal and cardiac tissue. Cardiomyocytes are post-mitotic, therefore post-translational effects of Cx43 better address the tissue environment^28,29^. One major contributor to stress signals in the DMD heart is oxidative stress^24,54,55^. In contrast, skeletal muscle is a syncytium in which fibers fuse and macrophages remain outside of the fibers. Insight for understanding the role of Cx43 may be further addressed by testing the response and modulation of reactive oxygen species in *mdx* versus WT macrophages. Data presented here suggest treatments limiting Cx43 and Cx43 hemichannel activity may also target Cx43+ skeletal muscle macrophages, which have roles in tissue repair and injury resolution. Macrophages have recently been shown to be essential for uptake and slow release of antisense oligonucleotides via Cx43^54,56,57^. The role as a reservoir is necessary for therapeutic administration of partially-functional dystrophin to skeletal fibers and muscle stem cell regeneration for DMD patients^39,43^. Cx43 is not enriched in *mdx* cardiac macrophages compared to the adjacent cardiomyocytes, reserving a larger role for Cx43 in the cardiomyocytes and skeletal-specific macrophages. We cannot rule out, however, a potential role for Cx43 in the cardiac macrophages, for example, at later stages of the disease.

Further investigation is necessary to determine if dystrophin from healthy fibers of a chimeric environment modulates Cx43 expression and function in neighboring macrophages, which in turn, modulate death of the neighboring fibers. In addition, we would like to investigate how this study translates to younger symptomatic carrier mice as we conducted studies in 6-month-old *mdx*/WT and *mdx*/WT-Cx43(+/−) chimeras with fibrosis in the former and no fibrosis in the latter (Supplementary Fig. S4).

As a limitation of the study, we cannot rule out an early developmental and postnatal contribution of Cx43 expression, which may still be highly expressed in the skeletal muscle, as the mixing of the WT blastocysts and the *mdx* ESCs takes place at pre-implantation stages of development. Thus, an early, developmental/postnatal effect of Cx43 may be a contributor to the long-term phenotype.

These results importantly contribute to research towards improved cardiomyopathic understanding and development towards target-specific (Cx43) skeletal muscle anti-inflammatory treatments for symptomatic DMD carriers.

## Methods

### Animals

WT (C57BL/6J), *mdx* (C57BL/10ScSn-Dmdmdx/J), and DsRed (B6.Cg-Tg(CAG- DsRed*MST)1Nagy/J), mice were purchased from Jackson Laboratories (Bar Harbor, ME, https://www.jax.org). *Chimeras*: Mouse and embryonic stem cell derivation and maintenance, and mouse chimera generation have been previously described in Gonzalez *et al*.^14^. We generated *mdx*/WT and *mdx*/WT-CX43(+/−) chimeras by injecting *mdx* ESCs into WT:Cx43(+/−) blastocysts. Quantification of ESC incorporation by genotyping of inserted genomic DsRed gene in *mdx* ESCs using primers fwd: 5′-GGTGATGTCCAGCTTGGAGT-3′ and rev: 5′-CCCCGTAATGCAGAAGAAGA-3′ (DsRed Jax) or previously published sequences^58^ and protocols^14^. Internal positive control primers provided by Jax Labs were used: fwd: 5′-CTAGGCCACAGAATTGAAAGATCT and rev: 5′-GTAGGTGGAAATTCTAGCATCATCC-3′. Chimeric and non-chimeric control animals were sacrificed and analyzed between 10–14 months (sample size has been incorporated into figures). A few chimeras were also sacrificed at 6 months of age. Chimeras were characterized and matched in pairs according to similar chimeric signatures determined by DsRed fluorescence from tail tip sections collected postnatally^14^ and DsRed genomic PCR of tissues collected at sacrifice (Figs. 1A and 2A, Supplementrary Fig. 3A), and dystrophin expression (Fig. S1A). Both males and females were combined due to the long history of the lab to obtain a lack of deviations at these time-points. Individual data points have been color-coded for transparency; red dots represent females and black dots represent males. All animal experiments were approved by the IACUC of Rutgers New Jersey Medical School and performed following relevant guidelines and regulations.

### Western blot

Western Blot analyses were previously described^14,24^. Following euthanasia in 10–14-month-old mice, tissues were rinsed in sterile PBS, flash-frozen in liquid nitrogen, and homogenized in RIPA buffer supplemented with protease and phosphatase inhibitors (Roche). Protein concentrations of tissue lysates were estimated using a BCA kit (Pierce). 15 μg protein (heart), or 30 μg protein (skeletal), per sample, was separated on 8% gels (Bio-Rad) 100–130 V for 90 minutes (heart), or 4–12% gradient gels (Bio-Rad) 100 V for 120 minutes (pectoralis, diaphragm). Gels were transferred to 0.45 µm nitrocellulose membrane 30 V for 16 hours at 4 °C. Ponceau S stain determined initial transfer success Blots were blocked in 5% non-fat laboratory-grade milk power (BioRad) in TBS-T. FACS cells were pelleted and lysed in RIPA buffer for 30 min with gentle agitation at 4 °C. Protein (normalized by cell number) approximated a volume relative to 60,000 macrophages was ran on a 10% gel for 100V-120V for 60–90 minutes. Gels were transferred to a 0.20 µm nitrocellulose membrane at 75 V for 2 hours. Probing steps were completed in 2% BSA. All gels were probed for Cx43 (Sigma, C6219, 1:10000), Dystrophin (Mandys8, 1:100, DSHB), GAPDH (Sigma, G9295, 1:10000), and Vinculin (1:2000, Sigma V9131). Blots were imaged and quantified using ECL reagent (Bio-Rad), Bio-Rad imaging box and image lab software.

### Immunofluorescence

Following euthanasia mouse hearts and skeletal muscle were harvested and rinsed in sterile PBS and frozen in Tissue-Tek^®^ OCT filled cryomolds using isopentane cooled on dry ice. Cryosections, 6 μm (heart) and 10 μm (skeletal muscle), were cut onto Precleaned Superfrost^®^ Plus slides (VWR Micro Slides) using Leica Cryostat with a −20 °C chamber. Sections were used immediately or stored at short-term(−20 °C) or long-term(−80 °C). Heart sections, stained for Cx43, were placed in chilled acetone for 10 minutes, washed, blocked in 10% Goat Serum in PBS-T (0.1% tween20 in 1X PBS) at room temperature (RT) and probed with the primary antibody Cx43 (1:1000, C6219, Sigma) and/or N-Cadherin (1:300, 33-3900, Invitrogen) overnight at 4 °C. Heart sections stained for dystrophin were thawed at RT for 30 minutes, washed with 1X PBS for 5 min, blocked for 1 hour in M.O.M.™ mouse IgG blocking reagent (BMK-2202, Vector M.O.M.™ immunodetection Kit, Vector Laboratories) and probed for using primary antibodies reactive to dystrophin (1:10, Mandys8, DSHB) in M.O.M.™ diluent overnight at 4 °C. All sections were washed 3 × 3 minutes in PBS-T (0.1% tween20 in 1X PBS) and probed with Alexa Fluor secondary antibodies (1:250, Invitrogen).

Skeletal muscle sections were thawed at RT and washed with 1X PBS. Sections, probed for Cx43 or F4/80, were fixed using 4% paraformaldehyde (PFA) for 10 minutes and then blocked for 1 hour in 10% Goat Serum in PBS-T at RT. Tissue sections were then probed with primary Cx43 (1:10,000, custom, generously supplied by Dr. Lampe) or F4/80 (1:100, MCA497RT, Bio-Rad). All sections were washed 3 × 3 minutes in PBS-T (0.1% tween20 in 1X PBS) and probed with Alexa Fluor secondary antibodies (1:250, Invitrogen). Cell borders were outlined with secondary conjugated WGA (1:500, W32464 Invitrogen, Thermo Fisher Scientific).

Afterward, muscle sections were washed in PBS-T and mounted using ProLong^TM^ Diamond antifade mountant with DAPI (P36962, Thermo Fisher Scientific). At least 3–5 sections (40x or 100x)were examined. Images were taken using a Nikon Eclipse T1 (Melville, NY, http://www.nikonusa.com) for immunoflourescence images, and an Olympus BX51 microscope for histological samples and processed using NIS-Elements BR software in a blinded fashion. Cross- sectional area (CSA) was measured using at least 200 fibers across 4–5 sections (100x and 200x). Colocalization was analysed using an Olympus Fluoview 1000 Confocal Laser Scanning Microscope and Z-stacks 0.5 µm thick at 600x magnification using Fluorview software and then processed in Fiji for maximum intensity. Cell counting and CSA were determined in Fiji in a blinded fashion.

### H&E and Masson trichrome

Skeletal and heart muscles were fixed in 4% paraformaldehyde overnight and embedded in paraffin, following euthanasia in 10–14-month-old mice. Sections were cut at 6 μm from each tissue and stained for histopathology utilizing an H&E kit (Vector, Torrance, CA) or Masson Trichrome kit (Richard-Allen Scientific, Thermo Fisher Scientific, Waltham, MA). Images were taken using a Nikon Eclipse T1 microscope and processed using NIS-Elements BR software. Fibrosis was quantified, blinded, by subtracting blue fibrotic regions from the total muscle area in Fiji and repeated in 5 images.

### Echocardiography

Animals were anesthetized with Avertin (290 mg/kg, IP) and maintained on a heated platform at 37 °C. Transthoracic echocardiography was performed using a 30-MHz ultrasound transducer (RMV707B) using VisualSonics Vevo 770 High-Resolution *In-Vivo* Micro-Imaging System. Mice, oriented in a shallow left lateral position, had warm coupling gel applied to the chest. Two-dimensional B-mode images and left ventricular M-mode tracings were acquired from the parasternal short-axis view at a sweeping speed of 100–200 mm/s at the level of the mid-papillary muscle. In a blinded fashion, M-mode measurements of left ventricular internal diameter (LVID) and wall thickness were acquired over eight consecutive beats and averaged using the leading-edge convention of the American Society of Echocardiography. Left ventricular ejection fraction (EF) was calculated following the cubed method using the following formula: EF(%) = 100 × [(LVIDd)^3^ − (LVIDs)^3^]/(LVIDd)^3^. Fractional Shortening (FS) was calculated following %FS = [LVIDd − LVIDs/LVIDd] × 100%. LVIDd: left ventricular end-diastolic dimension. LVID: left ventricular end-systolic dimension^59^. 2 echocardiographic determinations per mouse.

### Grip strength

An assessment of muscular function was recorded using a grip strength meter in a blinded fashion (Columbus Instruments). The grip strength meter was positioned horizontally, and mice, held by their tails, were lowered toward the pull bar. Mice (10–12 months) were allowed to get a good grip on the triangular bar with only their forelimbs. Once they had a good grasp, the mouse was pulled backward parallel to the device, until the mouse let go. This was completed in 2 runs: 5 pulls each. Run #1 trained and conditioned the mice to the apparatus. The top 3 scores from run #2 were averaged for the final value. This process was repeated 3-times over the 10–12 month period for each mouse, and the average used in statistical analysis. The force that was applied to the bar at the time of release was recorded as Gram-force.

### Fluorescent activated cell sorting (FACS)

Following euthanasia, diaphragms were dissected from 8–10-month-old WT and *mdx* mice, washed in 1% Pen/Strep in PBS, and minced. Digestion was completed using Liberase TL (Sigma) for 2 hours at 37 °C on an orbital shaker (20 rpm). Heart: the heart was dissected from 13–16-month-old *mdx* mice using a Lagendorff-free method^60^ describe below. Cells were filtered through a 40 μm cell strainer and incubated with antibodies F4/80, Cd11b and DAPI (Thermo Fisher Scientific) for 20 minutes on the benchtop covered from light. Samples were washed and sorted by the Flow Cytometry/Cell Sorting Rutgers Core Facility.

### Ethidium bromide uptake *in vitro*

Cells post-FACS-sort, from 8–10-month-old WT and *mdx* mice, were concentrated, mounted, and allowed to attach for 20-minutes on a chamber for confocal imaging. Ethidium Bromide (EthBr) was added during the first few frames of imaging (20 μM). A scanning confocal microscope (Olympus, FluoView1000) with a 600x objective lens captured an image every 10 seconds for 20 minutes (120 total images).

Gap19 Cx43 hemichannel-specific blocker (Sigma peptide mimetic) was added to chamber cells at the time of mounting for 20-minute incubation. While blinded to the treatment group, Fiji software was used to measure the fluorescence of a circle drawn around each cell in each frame (F). Values were normalized to the 3^rd^ frame (F_0_) after the addition of EthBr to account for variability induced by different rates of diffusion. Results are displayed as F/F_0_.

### Laggendorff-free isolation method for cardiac myocytes and nonmyocytes

The heart was dissected from 13–16-month-old *mdx* mice (N = 3). Single ventricular cardiomyocytes and mononuclear cells were isolated with Collagenase Type II (Worthington LS004176) and Protease XIV (Sigma P5147) by Laggendorf-free digestion of heart ventricle^60^. Myocyte and non-myocyte populations were separated by 20 minute of gravity filtration. Langendorff-free supernatant cell suspension was filtered through a 40 μm cell strainer and underwent the FACS preparation and sorting. The settled pellet of cardiomyocytes underwent two additional 15 minute gravity settlings in 4 mL of Perfusion buffer in a 15 mL tube^60^. The final pellet of myocytes was gently homogenized to obtain a protein sample used in western blot (Supplementary Fig. S4B,C).

### Statistics

Data was analyzed for statistical significance using parametric analysis in GraphPad Prism 8. Statistical significance was determined using one-way Brown-Forsythe and Welch analysis of variance statistical models (ANOVA) tests and Dunnett’s T3 multiple comparison test. Cross-sectional area (Fig. 2G) was assessed using Two-way ANOVA and Tukey’s multiple comparisons test. Alpha= 0.05. Two-tailed. Data are expressed as mean ± SEM. ^####^P < 0.0001, ^###^P < 0.0005, ^##^P < 0.005, ^#^P < 0.05 versus WT. ****P < 0.0001, ***P < 0.0005, **P < 0.005 *P < 0.05 versus mdx. ^$$$$^P < 0.0001, ^$$^P < 0.005, ^$^P < 0.05 for *mdx*/WT versus *mdx*/WT-Cx43(+/−) chimeras.

## Supplementary information

Supplementary information

## References

[1] Lapidos KA, Kakkar R, McNally EM (2004). The Dystrophin Glycoprotein Complex: Signaling Strength and Integrity for the Sarcolemma. Circ. Res..

[2] Moser H, Emery AEH (1974). The manifesting carrier in Duchenne muscular dystrophy. Clin. Genet..

[3] Soltanzadeh P (2010). Clinical and genetic characterization of manifesting carriers of DMD mutations. Neuromuscul. Disord..

[4] Lee SH, Lee JH, Lee KA, Choi YC (2015). Clinical and genetic characterization of female dystrophinopathy. J. Clin. Neurol..

[5] Song TJ, Lee KA, Kang SW, Cho H, Choi YC (2011). Three cases of manifesting female carriers in patients with duchenne muscular dystrophy. Yonsei Med. J..

[6] Politano L (1996). Development of cardiomyopathy in female carriers of Duchenne and Becker muscular dystrophies. J. Am. Med. Assoc..

[7] Juan-Mateu J (2012). Prognostic value of X-chromosome inactivation in symptomatic female carriers of dystrophinopathy. Orphanet J. Rare Dis..

[8] Papa R (2016). Genetic and Early Clinical Manifestations of Females Heterozygous for Duchenne/Becker Muscular Dystrophy. Pediatr. Neurol..

[9] Mercier S (2013). Genetic and clinical specificity of 26 symptomatic carriers for dystrophinopathies at pediatric age. Eur. J. Hum. Genet..

[10] Hoogerwaard EM (1999). Signs and symptoms of Duchenne muscular dystrophy and Becker muscular dystrophy among carriers in the Netherlands: A cohort study. Lancet..

[11] Watkins SC, Hoffman P, Slayter HS, Kunkel LM (1989). Dystrophin distribution in heterozygote mdx mice. Muscle & Nerve..

[12] Bostick B, Yue Y, Long C, Duan D (2008). Prevention of dystrophin-deficient cardiomyopathy in twenty-one-month-old carrier mice by mosaic dystrophin expression or complementary dystrophin/utrophin expression. Circ. Res..

[13] Bulfield G, Siller WG, Wight PA, Moore KJ (1984). X chromosome-linked muscular dystrophy (mdx) in the mouse. Proc. Natl. Acad. Sci..

[14] Gonzalez JP (2017). Small Fractions of Muscular Dystrophy Embryonic Stem Cells Yield Severe Cardiac and Skeletal Muscle Defects in Adult Mouse Chimeras. Stem Cells..

[15] Brioschi S (2012). Genetic characterization in symptomatic female DMD carriers: lack of relationship between X-inactivation, transcriptional DMD allele balancing and phenotype. BMC Med. Genet..

[16] Patrick Gonzalez J, Ramachandran J, Xie LH, Contreras JE, Fraidenraich D (2015). Selective Connexin43 Inhibition Prevents Isoproterenol-Induced Arrhythmias and Lethality in Muscular Dystrophy Mice. Sci. Rep..

[17] Lin LC (2005). Downregulated myocardial connexin 43 and suppressed contractility in rabbits subjected to a cholesterol-enriched diet. Lab. Investig..

[18] Schulz R (2015). Connexin 43 is an emerging therapeutic target in ischemia/reperfusion injury, cardioprotection and neuroprotection. Pharmacol. Ther..

[19] Ribeiro-Rodrigues TM, Martins-Marques T, Morel S, Kwak BR, Girão H (2017). Role of connexin 43 in different forms of intercellular communication – gap junctions, extracellular vesicles and tunnelling nanotubes. J. Cell Sci..

[20] Laird DW, Lampe PD (2018). Therapeutic strategies targeting connexins. Nat. Rev. Drug Discov..

[21] Delvaeye T, Vandenabeele P, Bultynck G, Leybaert L, Krysko DV (2018). Therapeutic Targeting of Connexin Channels: New Views and Challenges. Trends Mol. Med..

[22] Michela P, Velia V, Aldo P, Ada P (2015). Role of connexin 43 in cardiovascular diseases. Eur. J. Pharmacol..

[23] Hawat G, Hélie P, Baroudi G (2012). Single intravenous low-dose injections of connexin 43 mimetic peptides protect ischemic heart *in vivo* against myocardial infarction. J. Mol. Cell. Cardiol..

[24] Gonzalez JP (2018). Normalization of connexin 43 protein levels prevents cellular and functional signs of dystrophic cardiomyopathy in mice. Neuromuscul. Disord..

[25] Severs NJ, Bruce AF, Dupont E, Rothery S (2008). Remodelling of gap junctions and connexin expression in diseased myocardium. Cardiovasc. Res..

[26] Kleber AG, Saffitz JE (2014). Role of the intercalated disc in cardiac propagation and arrhythmogenesis. Front. Physiol..

[27] Wang N (2013). Selective inhibition of Cx43 hemichannels by Gap19 and its impact on myocardial ischemia/reperfusion injury. Basic Res. Cardiol..

[28] Lillo, M. A., Himelman, E., Xie, L.-H., Fraidenraich, D. & Contreras, J. E. S-Nitrosylation of Cx43 Hemichannels Promotes Cardiac Arrhythmias in a Duchene Muscular Dystrophy Mouse Model. *Biophys. J*. **4** (2019). 10.1172/jci.insight.130091 PMC6975272 31751316

[29] Himelman, E. *et al*. Prevention of Connexin43 remodeling protects against duchenne muscular dystrophy cardiomyopathy. *J. Clin. Invest*. (2020). 10.1172/JCI128190 PMC7108916 31910160

[30] Ishido, M. & Kasuga, N. Characteristics of the Localization of Connexin 43 in Satellite Cells during Skeletal Muscle Regeneration *In Vivo*. *Acta Histochem. Cytochem.* **48**, 53–60 (2015). 10.1267/ahc.14056 PMC4427565 26019374

[31] Cea LA (2013). De novo expression of connexin hemichannels in denervated fast skeletal muscles leads to atrophy. Proc. Natl. Acad. Sci..

[32] Kim Y (2016). Role of Hemichannels in CNS Inflammation and the Inflammasome Pathway. Adv. Protein Chem. Struct. Biol..

[33] Boengler K, Schulz R (2017). Connexin 43 and mitochondria in cardiovascular health and disease. Adv. Exp. Med. Biol..

[34] Cea LA (2016). Fast skeletal myofibers of mdx mouse, model of Duchenne muscular dystrophy, express connexin hemichannels that lead to apoptosis. Cell. Mol. Life Sci..

[35] Stedman HH (1991). The mdx mouse diaphragm reproduces the degenerative changes of Duchenne muscular dystrophy. Nature..

[36] Petrof BJ (2018). Diaphragm Weakness in the Critically Ill: Basic Mechanisms Reveal Therapeutic Opportunities. Chest..

[37] Li W (2018). Connexin 43 Hemichannel as a Novel Mediator of Sterile and Infectious Inflammatory Diseases. Sci. Rep..

[38] Dosch M (2019). Connexin-43-dependent ATP release mediates macrophage activation during sepsis. eLife..

[39] Dort, J., Fabre, P., Molina, T. & Dumont, N. A. Macrophages Are Key Regulators of Stem Cells during Skeletal Muscle Regeneration and Diseases. *Stem Cells Int*. 4761427 (2019). 10.1155/2019/4761427 PMC6664695 31396285

[40] Wang YX (2019). EGFR-Aurka Signaling Rescues Polarity and Regeneration Defects in Dystrophin-Deficient Muscle Stem Cells by Increasing Asymmetric Article EGFR-Aurka Signaling Rescues Polarity and Regeneration Defects in Dystrophin-Deficient Muscle Stem Cells by Increasin. Cell Stem Cell..

[41] Chen B, Shan T (2019). The role of satellite and other functional cell types in muscle repair and regeneration. J. Muscle Res. Cell Motility..

[42] Proulx A, Merrifield PA, Naus CCG (1997). Blocking gap junctional intercellular communication in myoblasts inhibits myogenin and MRF4 expression. Dev. Genet..

[43] Novak JS (2017). Myoblasts and macrophages are required for therapeutic morpholino antisense oligonucleotide delivery to dystrophic muscle. Nat. Commun..

[44] Araya R, Eckardt D, Riquelme M, Willecke K, Sáez J (2003). Presence and Importance of Connexin43 During Myogenesis. Cell Commun. Adhes..

[45] Merrifield PA, Laird DW (2016). Connexins in skeletal muscle development and disease. Semin. Cell Dev. Biol..

[46] Gorbe A, Krenacs T, Cook JE, Becker DL (2007). Myoblast proliferation and syncytial fusion both depend on connexin43 function in transfected skeletal muscle primary cultures. Exp. Cell Res..

[47] Retamal MA (2015). Diseases associated with leaky hemichannels. Front. Cell. Neurosci..

[48] Belousov AB, Fontes JD, Freitas-Andrade M, Naus CC (2017). Gap junctions and hemichannels: Communicating cell death in neurodevelopment and disease. BMC Cell Biol..

[49] Cea LA (2016). Dexamethasone-induced muscular atrophy is mediated by functional expression of connexin-based hemichannels. Biochim. Biophys. Acta - Mol. Basis Dis..

[50] Helming L, Gordon S (2009). Molecular mediators of macrophage fusion. Trends Cell Biol..

[51] Saclier M (2013). Differentially activated macrophages orchestrate myogenic precursor cell fate during human skeletal muscle regeneration. Stem Cells..

[52] Xing LY, Yang T, Cui SS, Chen G (2019). Connexin hemichannels in astrocytes: Role in CNS disorders. Front. Mol. Neurosci..

[53] Jara PI, Boric MP, Sáez JC (1995). Leukocytes express connexin 43 after activation with lipopolysaccharide and appear to form gap junctions with endothelial cells after ischemia-reperfusion. Proc. Natl. Acad. Sci. USA.

[54] Anand RJ (2008). A role for connexin43 in macrophage phagocytosis and host survival after bacterial peritoneal infection. J. Immunol..

[55] Allen DG, Whitehead NP, Froehner SC (2015). Absence of Dystrophin Disrupts Skeletal Muscle Signaling: Roles of Ca 2+, Reactive Oxygen Species, and Nitric Oxide in the Development of Muscular Dystrophy. Physiol. Rev..

[56] Novak JS, Jaiswal JK, Partridge TA (2018). The macrophage as a Trojan horse for antisense oligonucleotide delivery. Expert Opin. Ther. Targets..

[57] Yue Y, Liu M, Duan D (2006). C-Terminal-Truncated Microdystrophin Recruits Dystrobrevin and Syntrophin to the Dystrophin-Associated Glycoprotein Complex and Reduces Muscular Dystrophy in Symptomatic Utrophin/Dystrophin Double-Knockout Mice. Mol. Ther..

[58] Shin JH, Hakim CH, Zhang K, Duan D (2011). Genotyping mdx, mdx3cv, and mdx4cv mice by primer competition polymerase chain reaction. Muscle and Nerve..

[59] Gao S, Ho D, Vatner DE, Vatner SF (2011). Echocardiography in Mice. Curr. Protoc. Mouse Biol..

[60] Ackers-Johnson M (2016). A Simplified, Langendorff-Free Method for Concomitant Isolation of Viable Cardiac Myocytes and Nonmyocytes from the Adult Mouse Heart. Circ. Res..

