# Supplementary material for: Connexin-43 reduction prevents muscle defects in a mouse model of manifesting Duchenne muscular dystrophy female carriers
Source: Sci Rep. 2020 Mar 30;10:5683. doi: 10.1038/s41598-020-62844-9 (PMC7105483; doi:10.1038/s41598-020-62844-9)
Supplement: Supplementary file 1 — Supplementary information [file 41598_2020_62844_MOESM1_ESM.pdf]

Supplementary Information

Connexin-43 reduction prevents muscle defects in a mouse model of manifesting Duchenne muscular dystrophy female carriers.

Julie Nouet, Eric Himelman, Kevin C. Lahey, Qingshi Zhao and Diego Fraidenraich*

Department of Cell Biology and Molecular Medicine, Rutgers Biomedical and Health Sciences, New Jersey Medical School, Newark, NJ USA

*To whom correspondence should be addressed: fraidedi@rutgers.edu

Figure S1

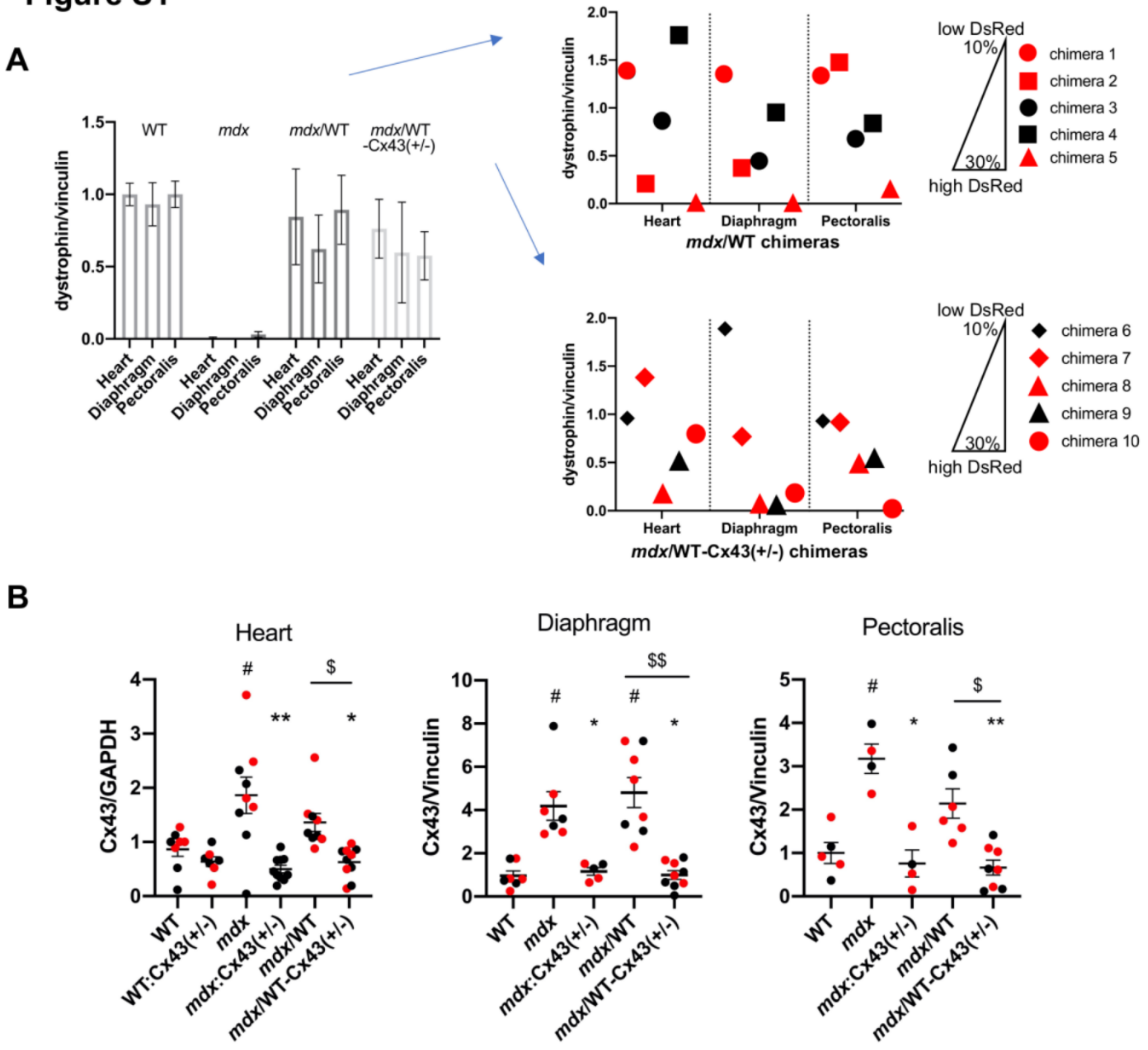

Figure S1. Dystrophin and Cx43 quantification in individual chimeras. (A) Left panel: dystrophin in heart, diaphragm, and pectoralis for WT, *mdx* and *mdx*/WT and *mdx*/WT-Cx43(+/-) chimeras. Values are relative to dystrophin levels assessed in heart (1.0). Top right: protein expression levels via western blot analysis plotted in *mdx*/WT individual chimeras for heart, diaphragm, and pectoralis (chimeras 1-5). Bottom right: protein expression levels via western blot plotted in *mdx*/WT-Cx43(+/-) individual chimeras for heart, diaphragm, and pectoralis (chimeras 6-10). Degree of chimerism (10-30%) was determined by DsRed tail immunofluorescence at birth and DsRed genomic PCR of tissues at sacrifice (see Methods). Note variation between tissues (heart, diaphragm and pectoralis) between chimeras, due in part by the patchy character of ESC incorporation. **(B)** Cx43 protein expression quantified from western blot in heart, diaphragm, and pectoralis. Statistical analysis was completed using Brown-Forsythe and Welch ANOVA tests and Dunnett's T3 multiple comparison test. Data are presented as means ± SEM. ; # $p<0.05$ versus WT. ** $p<0.005$, * $p<0.05$ versus mdx. $ $p<0.05$, $$ $p<0.005$ *mdx*/WT versus *mdx*/WT-Cx43(+/-) chimeras. Red dots represent female mice and black dots represent male mice. Mouse age range: 10-14 months.

Figure S2

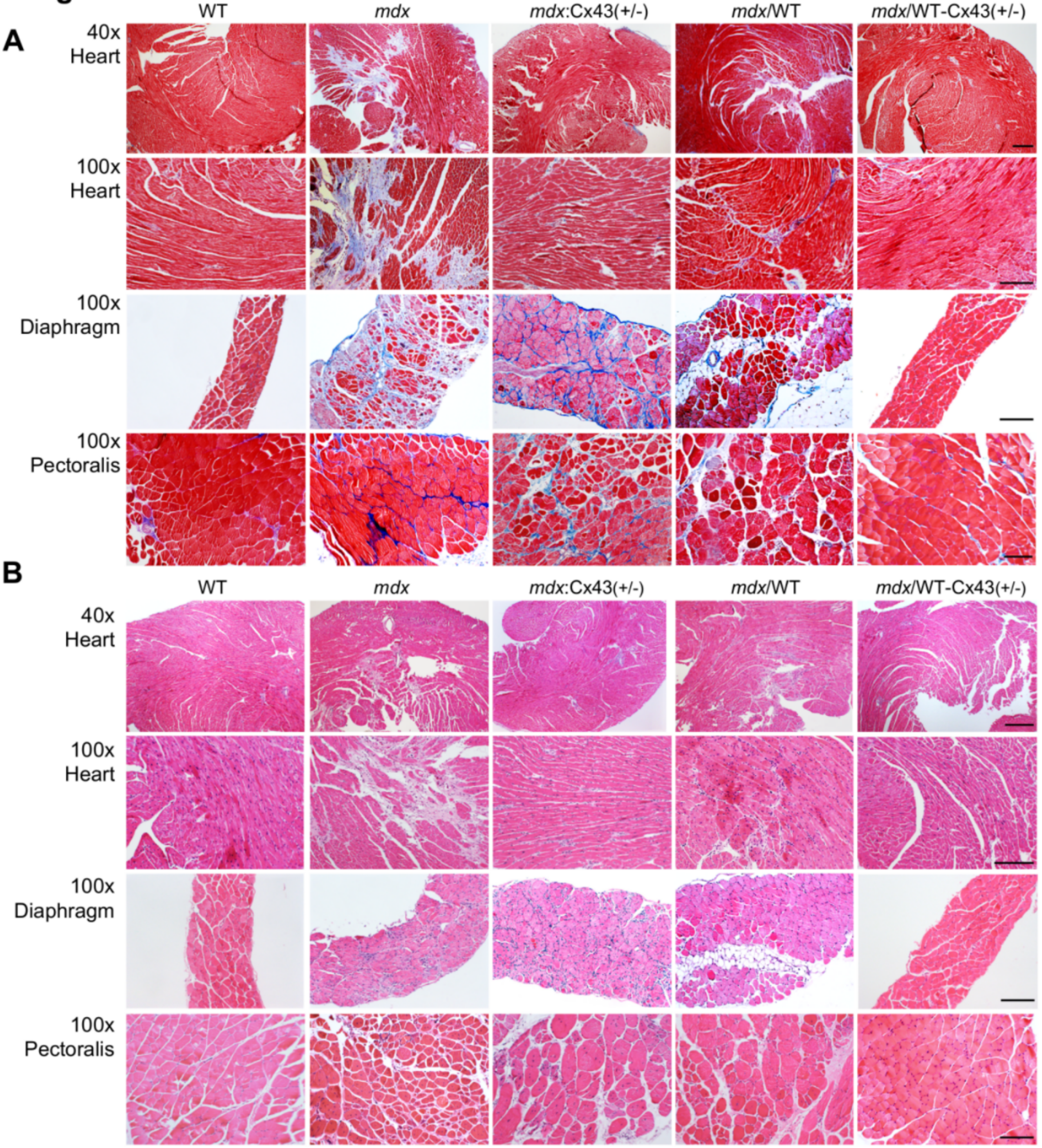

Figure S2. Histological images of heart, diaphragm and pectoralis muscle at low magnification. Representative Masson trichrome (MT) **(A)** and H&E **(B)** images show fibrosis (MT), mononuclear invasion (H&E) and central nucleation (H&E) in *mdx*, *mdx*:Cx43(+/-) mice and *mdx*/WT chimeras but not in WT mice and *mdx*/WT-Cx43(+/-) chimeras. Magnification: 40X (heart) and 100X (heart, diaphragm and pectoralis). Scale bar: 150 mm. Mouse age range: 10-14 months.

Figure S3

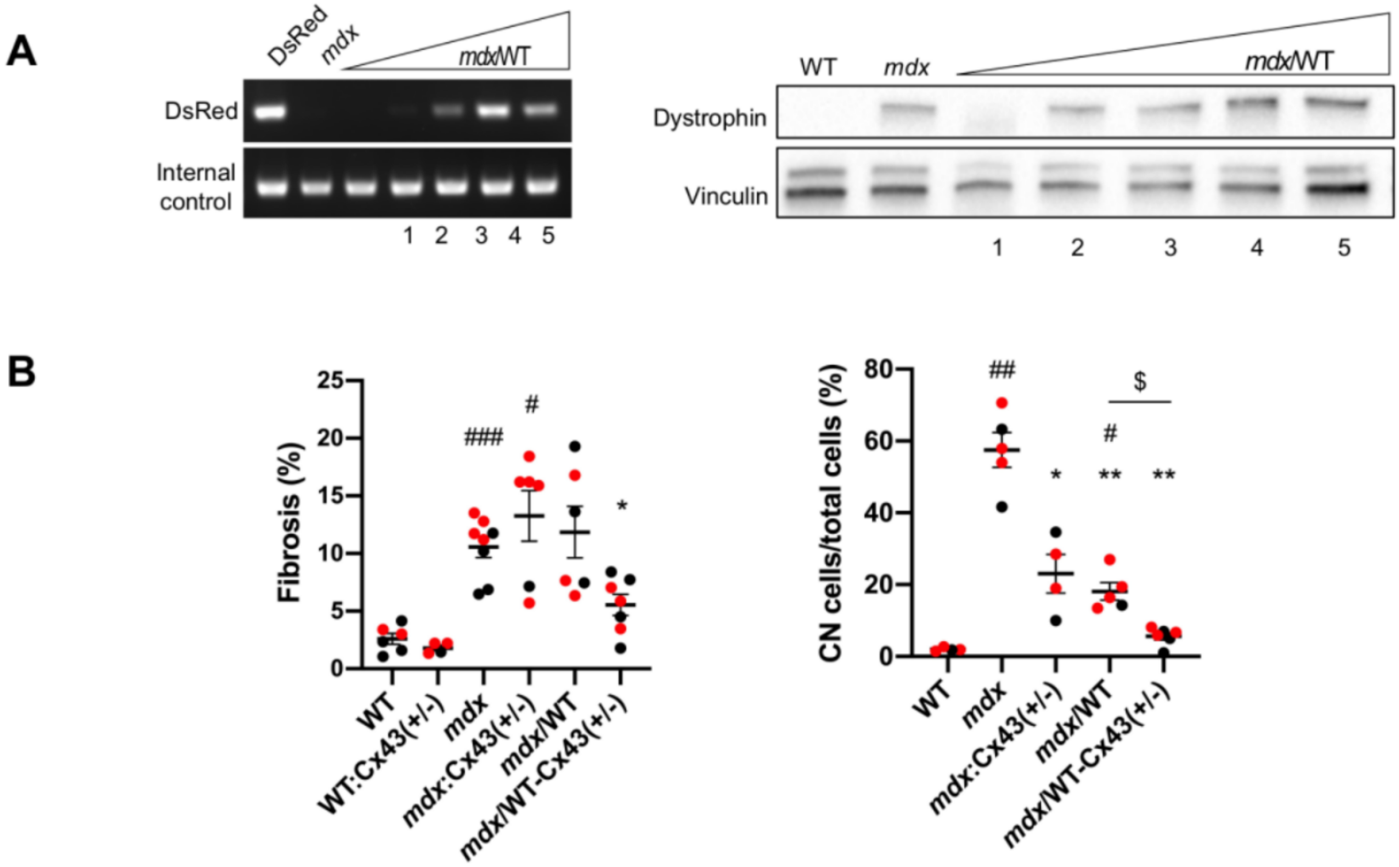

Figure S3. Percentage of fibrosis and central nucleation of pectoralis muscle. (A) Left panel: A representative agarose gel shows PCR products of the DsRed transgene (top band) from pectoralis genomic DNA of DsRed (100% control), *mdx* (0% control) and *mdx*/WT chimeras with increasing degrees of chimerism (left to right). Internal control (bottom band) serves as normalizer. Right panel**:** A representative western blot stained for dystrophin (top band), using pectoralis protein extracts of *mdx* (0% control), WT (100% control), and *mdx*/WT chimeras with increasing levels of dystrophin (left to right). Vinculin (bottom band): internal control. Samples presented in A (1-5) and B (1-5) are paired. **(B)** Left panel: Fibrosis (%). Sample size: WT, WT:Cx43(+/-), *mdx*, *mdx*:Cx43(+/-), *mdx*/WT, *mdx*/WT-Cx43(+/-) respectively left to right N(6,4,8,6,6,7). Right panel: percent (%) central nucleation (CN) of pectoralis tissue. Sample size: WT, *mdx*, *mdx*:Cx43(+/-), *mdx*/WT, *mdx*/WT-Cx43(+/-) respectively left to right N(4,5,4,5,6). Statistical significance was determined using Brown-Forsythe and Welch ANOVA tests and Dunnett's T3 multiple comparison test. Data are presented as means ± SEM. ; ### p<0.0005, ## p<0.005, # p<0.05 versus WT. ** p<0.005, * p <0.05 versus mdx. $ p<0.05 *mdx*/WT versus *mdx*/WT-Cx43(+/-) chimeras. Red dots represent female and black dots represent male mice. Mouse age range: 10-14 months. Uncropped gel and blot are displayed in figure S6.

Figure S4

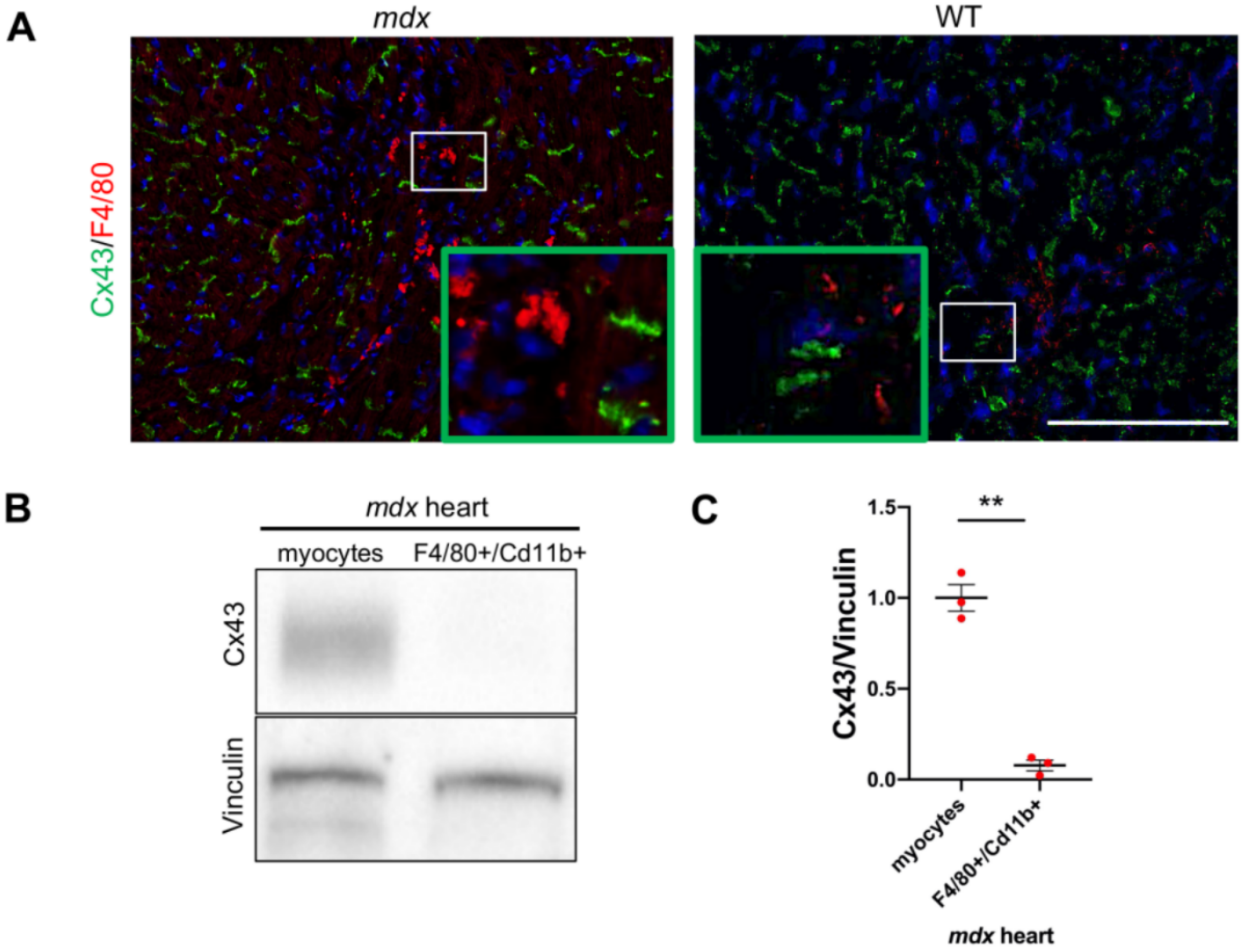

Figure S4. Cx43 is detected in dystrophic cardiomyocytes but not in cardiac macrophages. (A) Representative immunofluorescent images do not show overlap of Cx43 (green) and F4/80 (red), in cryosections of *mdx* or WT heart muscle. DAPI (blue) demarcates nuclei. Magnification: 200x. Scale Bar: 150 mm. **(B)** Representative western blot for Cx43 (top row) and loading control Vinculin (bottom row) in gravity-isolated mdx cardiomyocytes and FACS-isolated F4/80+/Cd11b+ heart mdx macrophages. **(C)** Quantification of Cx43 expression in **B** (N=3 mice). Statistical significance was determined by Brown-Forsythe and Welch ANOVA tests. Data are presented in means ± SEM; ** p<0.005 versus *mdx*. Red dots represent female mice and black dots represent male mice. Mouse age range: 13-16 months. Uncropped blots are displayed in figure S6.

Figure S5

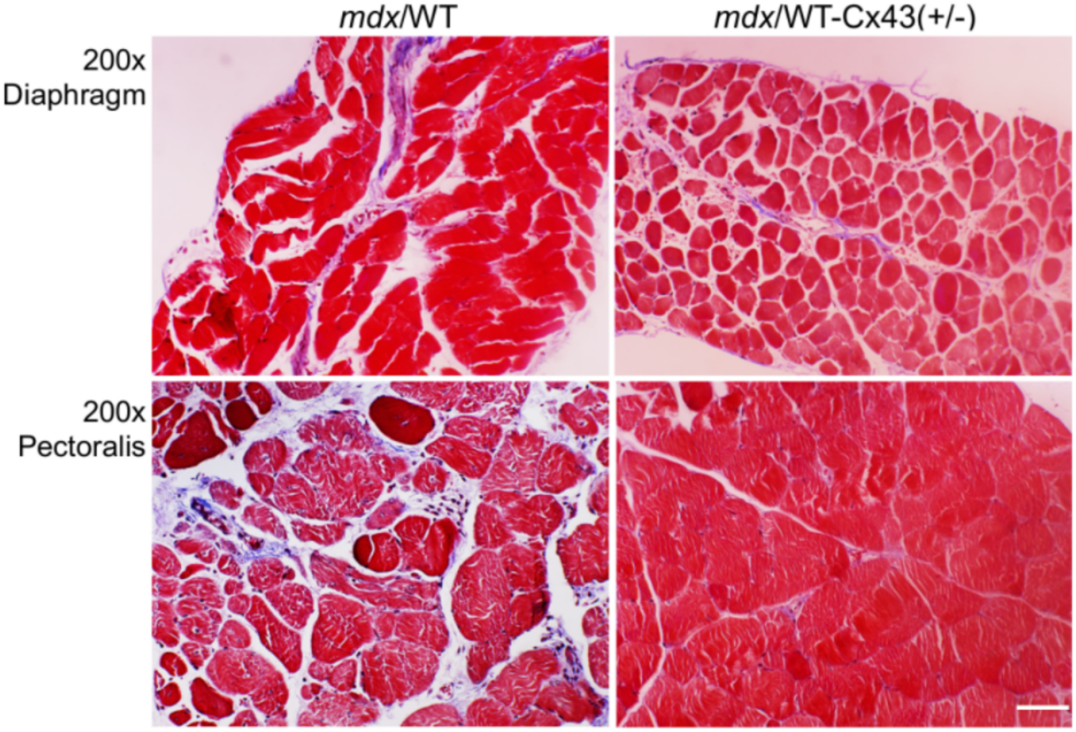

Figure S5. Masson trichrome in 6 month-old *mdx*/WT and *mdx*/WT-Cx43(+/-) chimera diaphragm and pectoralis. Representative Masson trichrome (MT) images show fibrosis (in *mdx*/WT chimera pectoralis but not in *mdx*/WT-Cx43(+/-) chimera pectoralis. Representative photos at 200x magnification. Scale Bar: 150 mm. *mdx*/WT: N=2; *mdx*/WT-Cx43(+/-): N=3.

Figure S6

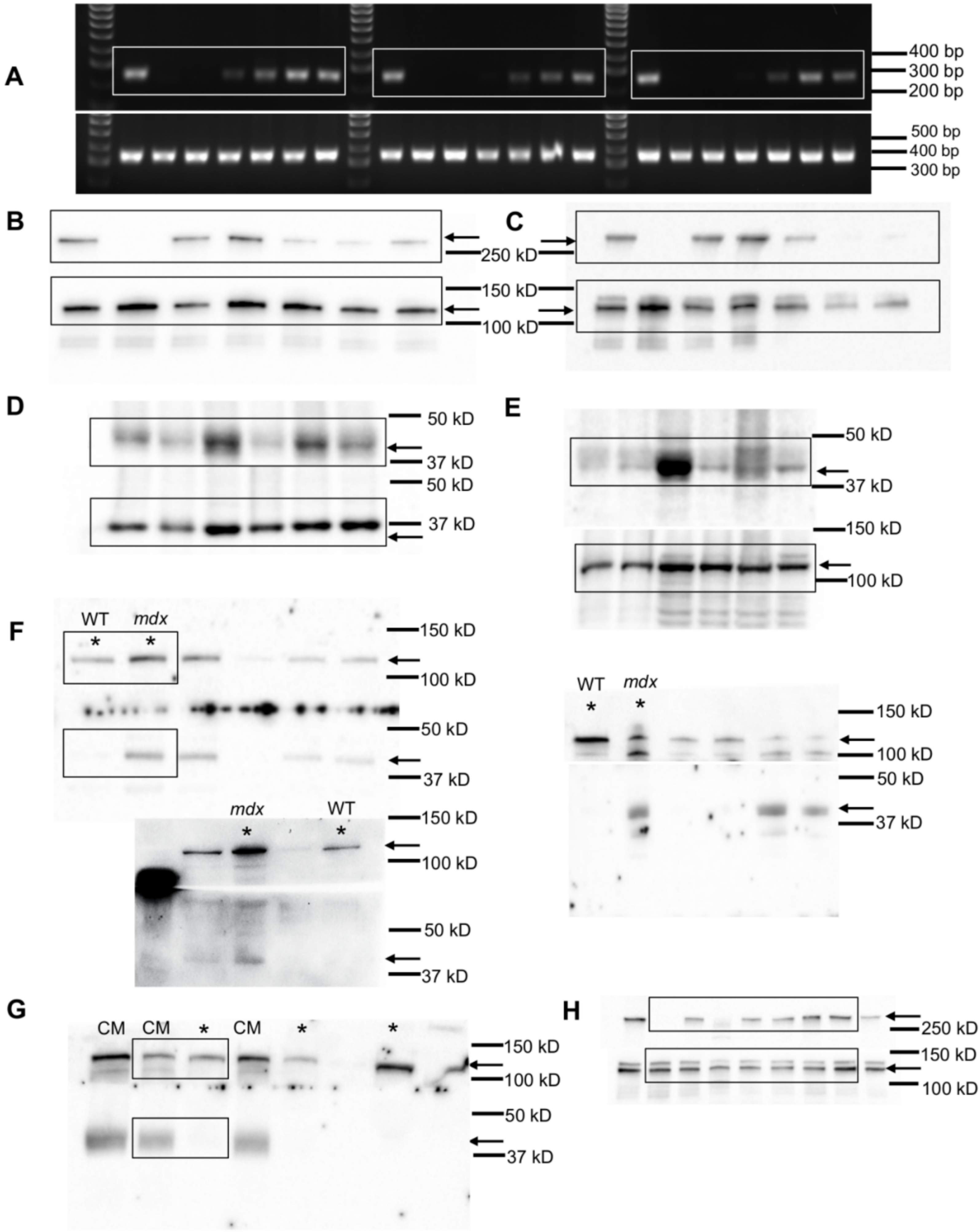
A
400 bp
300 bp
200 bp
500 bp
400 bp
300 bp
B
250 kD
150 kD
100 kD
C
D
50 kD
37 kD
50 kD
37 kD
E
50 kD
37 kD
150 kD
100 kD
F
WT
mdx
*
150 kD
100 kD
50 kD
37 kD
mdx
WT
150 kD
100 kD
50 kD
37 kD
G
CM
CM
*
CM
*
*
150 kD
100 kD
50 kD
37 kD
H
250 kD
150 kD
100 kD

Figure S6. Full-length uncropped PCR gels and western blots. (A) PCR of DsRed (top) and internal control (bottom). Heart (left) in Figure 1A, diaphragm (right) in Figure 2A, and pectoralis (center) in Figure S3a left. **(B)** Dystrophin (top arrow) and Vinculin (bottom arrow) in Figure 1B. **(C)** Dystrophin (top arrow) and Vinculin (bottom arrow) in Figure 2B. **(D)** Cx43 (top arrow) and GAPDH (bottom arrow) in Figure 1D. **(E)** Vinculin (top arrow) and Cx43 (bottom arrow) in Figure 2C. **(F)** Vinculin (top arrow) and Cx43 (bottom arrow). Top Left blot is displayed in Figure 3E. WT*: WT F4/80+/Cd11b+ FACS-macrophages. *mdx**: *mdx* F4/80+/Cd11b+ FACS-macrophages from diaphragms. Bottom left blot and right blots represent additional samples used for quantification in Figure 3F. **(G)** Vinculin (top arrow) and Cx43 (bottom arrow) in Figure S4B. Labels: CM (cardiomyocytes) and * (mdx heart F4/80+/Cd11b+ FACS-macrophages). **(H)** Dystrophin (top arrow) and Vinculin (bottom arrow) in Figure S3b. All gels and blots: Boxes demarcate images shown in the figure panels.
